# Fertility Concerns Related to Surgery for Colorectal Cancer: An Under-Discussed Topic

**DOI:** 10.3390/cancers16193376

**Published:** 2024-10-02

**Authors:** Samantha L. Savitch, Maedeh Marzoughi, Pasithorn A. Suwanabol

**Affiliations:** 1 Department of Surgery, University of Michigan Health System, Ann Arbor, MI 48109, USA; pasuwan@med.umich.edu; 2 University of Michigan Medical School, Ann Arbor, MI 48109, USA; maedeh@med.umich.edu; 3 Center for Healthcare Outcomes and Policy, University of Michigan, Ann Arbor, MI 48109, USA

**Keywords:** colorectal cancer, infertility, counseling, colorectal surgery, fertility preservation

## Abstract

**Simple Summary:**

As the incidence of colorectal cancer (CRC) increases among younger adults, the need for discussions regarding treatment-related infertility is growing. Treatment-related infertility is distressing to patients, and data show that patients desire pre-treatment fertility counseling and would take advantage of opportunities for fertility preservation. That said, counseling occurs far too infrequently, and surgeons are vastly underutilized for these conversations. In this review, we highlight the current knowledge regarding infertility and counseling in patients undergoing CRC treatment and provide an overview of the connection between abdominal and pelvic surgery on male and female infertility. Further, we identify topics of importance when conducting infertility counseling and elucidate the role of surgeons in these important conversations.

**Abstract:**

As the incidence of colorectal cancer (CRC) increases among younger adults, the need for discussions regarding treatment-related infertility is growing. The negative impacts of gonadotoxic chemotherapy and pelvic radiation are well documented, but the role that surgical intervention for CRC plays in infertility is less clear. Additionally, treatment-related infertility counseling occurs infrequently. This review provides an overview of the connection between abdominal and pelvic surgery on male and female infertility and elucidates the role of surgeons in counseling to alleviate psychological distress in newly diagnosed patients. A review of the literature revealed that pelvic surgery leads to increased adhesion formation, which is known to be associated with female infertility. Furthermore, nerve damage from pelvic surgery has significant implications for ejaculatory issues in males and sexual dysfunction in both males and females, which ultimately impact pregnancy success. Patients have significant distress related to treatment-related infertility, and pre-treatment fertility counseling has been shown to alleviate some of this psychological burden. Nevertheless, many patients do not receive counseling, particularly in surgical clinics, despite surgeons often being the first providers to see newly diagnosed non-metastatic patients. Efforts should be made to enact protocols that ensure fertility conversations are being had with patients in surgical clinics and that patients are being referred to fertility specialists appropriately. This patient-centered approach will lessen the psychological burden placed on patients during a vulnerable time in their lives.

## 1. Introduction

The incidence of colorectal cancer (CRC) in younger adults is rising exponentially, with an expected increase of greater than 100% by 2030 [1]. The American Cancer Society estimates that each year in the U.S., 13% of CRC diagnoses are among those less than 50 years of age (i.e., early-onset CRC) [2]. As the number of individuals with early-onset CRC grows, concerns over fertility and sexual health are increasingly being brought to the forefront given that these patients are at the height of their reproductive potential. Yet, less than two thirds of younger adults diagnosed with CRC are even aware that treatment can pose a risk to future fertility [3], and far fewer have documented fertility discussions with their providers [4].

Studies have shown that females who develop CRC have decreased fertility compared to females who do not develop CRC [5]. Most of the infertility risk is associated with gonadotoxic chemotherapy and pelvic radiation, with a paucity of data focused on the impact of surgery specifically. That said, research performed in patients undergoing colorectal surgery for non-malignant disease indicates significant impacts on both male and female fertility. However, surgeons are the initiators of fertility discussions less than 25% of the time [6], despite these providers being some of the first to see patients after an initial diagnosis of CRC.

In this review, we highlight the current knowledge regarding infertility and counseling in patients undergoing CRC treatment and will provide an overview of the connection between abdominal and pelvic surgery on male and female infertility. Further, we will discuss the psychological impact that treatment-related infertility has on patients, as well as how patient-centered conversations can relieve stress and improve the patient–provider relationship. Finally, we will elucidate the role of surgeons in these important conversations.

## 2. The Role of Surgery in Infertility

While the vast majority of research focused on fertility in patients with CRC has examined the impacts of chemotherapy and radiation, there are some studies focused on the role that surgical intervention plays. From the existing literature, we now appreciate that some patients with early-onset CRC who undergo surgical resection experience sexual dysfunction (6%) and infertility (1%). Though the underlying mechanisms have yet to be determined [7], it has been hypothesized that adhesions, direct damage to sexual organs, and nerve damage may be the cause [8].

Relative to the data demonstrating infertility among those with inflammatory bowel disease (IBD) and familial adenomatous polyposis (FAP), infertility among those with CRC is less well established. However, from studies focused on benign conditions of the colon, much light can be shed on the likely impact that surgery is having in cancer patients. In patients with both ulcerative colitis (UC) and FAP, multiple studies have shown that fertility rates are decreased after surgical intervention [9,10], with one study showing a relative risk of infertility of 3.17 after ileal pouch anal anastomosis (IPAA) as compared to medical management of UC [10]. In patients with IBD, the risk of infertility is higher after IPAA compared with non-restorative proctectomy [10,11,12], which is likely because rectal resection is more damaging to pelvic nerves and pelvic organs. However, there is a lack of consensus regarding the impact on infertility of ileorectal anastomosis (IRA) compared to IPAA in patients with FAP. One study performed in patients with FAP showed no difference between IRA and IPAA, though younger patients had a higher incidence of fertility issues [13]. Conversely, Olsen et al. show that patients who undergo IRA have similar fertility to the general population, while those who undergo IPAA experience a significant decrease in fertility to 54% [11]. Importantly, for those who undergo IPAA and later pursue in vitro fertilization (IVF), IVF success rates are similar to patients who do not undergo IPAA [14].

The prevailing theory regarding female infertility after pelvic surgery is that it is due to adhesive disease, which alters the normal anatomic relation of the ovaries, fimbriae, and fallopian tubes [15,16,17]. Bowman et al. evaluated follicle-stimulating hormone (FSH) levels in 66 patients undergoing laparoscopy for infertility workup and showed decreased FSH levels in patients with adhesive disease [18], suggesting a link between adhesions and ovarian function. Postoperative adhesions and scarring can also lead to loss of fallopian tube patency [19]. Similarly, damage can occur to the fallopian tubes, ovaries, and fimbriae directly during pelvic dissection.

For surgeons determining ways to decrease female fertility risks intra-operatively, current data suggest that laparoscopic approaches decrease overall [20,21,22] and adnexal adhesions [23,24,25]. Hull et al. examined 40 patients with UC undergoing diagnostic laparoscopy at the time of ileostomy closure and found significantly lower adnexal adhesion scores in patients undergoing laparoscopic as compared to open IPAA [23]. In some studies, fertility rates have not been shown to be different between patients undergoing laparoscopic and open surgery, though time to pregnancy is decreased in patients who underwent laparoscopic approaches [26]. Others have shown that patients undergoing laparoscopic IPAA have lower infertility rates than those undergoing open surgery [27]. As a result, particularly for patients who wish to maintain post-treatment fertility, efforts should be made to pursue minimally invasive approaches for patients with a favorable risk profile.

It is also important to acknowledge the impact of surgical interventions on sexual dysfunction and the role that sexual dysfunction plays in fertility, particularly in male patients. Over 50% of patients report sexual dysfunction after abdominopelvic surgery [28], which is generally thought to be due to nerve damage. As such, it is more common in low pelvic operations due to proximity of the autonomic nerves associated with sexual function and is not as commonly encountered in patients undergoing colon resections proximal to the sigmoid colon [29] (see Table 1). In males, sexual dysfunction presents most commonly as erectile dysfunction and retrograde ejaculation [8,29,30,31], generally due to nerve damage. Nishizawa et al. performed a prospective study of 207 patients undergoing total mesorectal excision (TME) for low rectal cancer and showed that, of the patients who were sexually active, 82% developed post-operative ejaculatory problems [32]. This is consistent with other studies reporting that erectile dysfunction and ejaculatory issues are common after pelvic surgery [8,28,29,30,31], supporting the need for preoperative sperm preservation among male patients who are interested in having children in the future.

## 3. The Impacts of Chemotherapy and Radiation on Fertility

While not the primary focus of this review, the effect of chemotherapy and radiation therapy on fertility should be briefly discussed. Chemotherapeutic regimens for patients with CRC are usually 5-flurouracil (5-FU) based, and while 5-FU is not a particularly gonadotoxic therapy, there have been reports of patients having fertility issues after undergoing treatment with 5-FU [36]. Commonly, 5-FU is combined with oxaliplatin, which is known to have moderate gonadotoxic effects [37]. Cercek et al. show that 16% of women under the age of 50 years who receive FOLFOX (folinic acid, 5-FU, and oxaliplatin) experience amenorrhea post-treatment [38]. Furthermore, the addition of antiangiogenic agents, such as bevacizumab, may increase the fertility risk even further, with one study showing 34% ovarian failure rate in patients receiving bevacizumab and FOLFOX compared with 2% ovarian failure in patients receiving FOLFOX alone [38]. In this study, approximately 20% of patients who receive bevacizumab recover ovarian function after cessation of the drug.

Studies show that premature ovarian failure, defined as undetectable anti-Mullerian hormone (AMH) levels, occurs in over 90% of patients who undergo full-dose (45–50 Gy) radiation therapy for rectal cancer [37,39]. Wan et al. show that among 123 premenopausal women under the age of 40 years, only 4% of patients with colon cancer experience persistent amenorrhea compared with 94% of patients with rectal cancer [40], likely due to the use of pelvic radiation in the rectal cancer cohort.

## 4. Options for Fertility Preservation

Fertility preservation procedures are more complicated for females than males, but there are more options for female patients than for their male counterparts. For males, the primary method of fertility preservation is sperm banking [41], though there are options for post-operative therapies to improve ejaculatory function that may be outside the scope of this review. For females, the first-line method of preservation is oocyte or embryo cryopreservation. This approach requires an approximate 2-week delay prior to initiating chemotherapy [37], which can add significant stress to patients as they cope with a new diagnosis and the time constraints of this invasive procedure [42,43]. This can also create tension between the priorities of patients and providers, as studies have shown that patients sometimes feel that their healthcare providers prioritize starting treatment over preserving fertility [44]. In select cases, patients may have reason to start treatment urgently, such as impending or active obstruction, significant gastrointestinal bleeding, or other emergent issues. It should be noted that the majority of patients can safely postpone the initiation of treatment in favor of oocyte or embryo cryopreservation if desired.

Other options for fertility preservation include oophoropexy, wherein the ovaries are repositioned above the pelvic brim to shield them from radiation scatter [37]. It is important to note that while this decreases the effect of radiation therapy on ovarian function, it does not offer complete protection, with some studies showing a 50% 5-year ovarian survival after the procedure [45]. Patients should thus also be offered oocyte or embryo preservation. Oophoropexy can be performed at the time of cancer resection; as such, it is important that conversations about fertility preservation occur prior to surgical resection, as performing oophoropexy at the same time as resection negates the need for a second surgical procedure, which may be more complicated if adhesions form.

Regardless of whether patients undergo fertility preservation procedures, it is recommended that female patients who desire post-treatment fertility undergo baseline AMH testing, as these levels can be tracked post-treatment as a marker of long-term fertility [37]. Some authors have recommended waiting two years post-treatment to start trying to conceive, as the majority of CRC recurrences occur in the first two years after diagnosis [37].

## 5. The Psychologic Burdens of Treatment-Related Infertility and the Role of Fertility Counseling

Though not evaluated in colorectal cancer specifically, numerous studies have been performed in adolescent and young adult patients with other malignancies assessing the impact of cancer treatment-related infertility on psychological well-being. These studies have shown that both male and female patients feel distress and anxiety related to infertility associated with their cancer diagnosis and treatment [42,46,47]. Specifically, patients express feeling “troubled” when their concerns about fertility are poorly managed [48], and they feel particularly burdened when the onus falls on them to bring the conversation up with their providers [49]. For providers, presenting newly diagnosed cancer patients with their treatment options and focusing on initiating treatment is often paramount, but this leads to patient dissatisfaction and feelings of lack of control when patients have fertility concerns and can ultimately sour the patient–provider therapeutic relationship [43,50]. On the other hand, patients can be overwhelmed when presented with a cancer diagnosis and may not be aware of fertility risks or may not have considered their desire for future fertility previously [42,43,51,52]. It is therefore the responsibility of the provider to initiate these conversations so that patients have the time to plan for future fertility, should they desire it.

While the prospect of infertility can be psychologically burdensome, research shows that counseling about fertility preservation offers patients a sense of control and hope and improves the overall quality of life [46,51,53,54]. The option of fertility preservation also alleviates the stress related to future infertility so that patients have the mental space to focus on their cancer treatment [43]. Both male and female patients report that the option of fertility preservation is important to them, even though the process can be uncomfortable and stressful [41,46,54,55,56]. Furthermore, data show that fertility counseling increases the utilization of fertility preservation procedures, especially sperm banking [57].

It is reported that up to 75% of adolescent and young adult cancer survivors are interested in starting families at some point, though a significantly lower proportion of these individuals take advantage of fertility preservation services [58]. Much of this discrepancy is due to the underutilization of pre-treatment fertility counseling, with some studies showing that up to 75% of patients are receiving some sort of pre-treatment counseling [59,60], whereas others cite far more concerning rates of 15–20% [61,62]. Despite the American Society for Clinical Oncology (ASCO) publishing guidelines since 2006 supporting fertility counseling for all cancer patients of reproductive age [63], as well as the National Comprehensive Cancer Network (NCCN) guidelines recommending fertility counseling [64,65], data show that the frequency of these conversations has not increased [66]. Stal et al. performed a survey study of 148 rectal cancer survivors who were diagnosed prior to age 50 and found that over half of patients did not have any sort of pre-treatment fertility discussions [67]. Furthermore, only one fifth of the patients underwent fertility preservation such as sperm, oocyte, or embryo preservation. Others have found similarly low rates of fertility preservation, with some patients stating that insurance presents a large barrier to pursuing these interventions [59]. In fact, patients living in areas of relative poverty are less likely to receive fertility counseling, both due to the limited availability of counseling services and the high out of pocket costs [68]. Low referral rates may also be a contributor to the underutilization of fertility preservation [6].

Factors that increase the likelihood of fertility discussions include older age, greater quality of life, rectal cancer, nulliparity, and the need for radiation treatment [6,59,67], though even patients with these characteristics are not consistently receiving proper counseling. While around 50% of patients report having some sort of conversation regarding reproductive health, fertility preservation specifically is rarely discussed [6]. Importantly, evidence shows that patients care about these conversations, with many patients reporting after the fact that they wished they had received counseling [6]. Providers may be hesitant to bring up these discussions when they believe patients are not candidates for fertility preservation procedures; however, it is recommended that fertility risks be discussed even in this population, so that patients are not blindsided in the future [46]. Furthermore, while fertility preservation may be cost-prohibitive, infeasible, or not desired by some patients, studies show that simply receiving counseling on infertility risks and fertility preservation options can give patients hope and a sense of control [46,69,70]. Importantly, survey studies indicate that patients wish they had been told about the risks of infertility and the option of fertility preservation prior to initiating treatment [6,46,67,71].

Per NCCN guidelines, surgical resection is the primary treatment modality for non-metastatic colon and rectal cancer [64,65]. As such, surgeons may be one of the first providers to have prolonged interactions with newly diagnosed patients. Unfortunately, data show that surgeons are initiating these conversations only 22% of the time, highlighting an area of opportunity for improvement [6]. One of the difficulties that may underlie these numbers is a lack of guidance regarding what should be discussed and how to discuss it with patients, and the absence of quality information and support from providers can augment patient unease regarding future infertility [46,48]. ASCO and the European Society for Medical Oncology (ESMO) recommend that the following should be included in any fertility counseling conversation [63,72]:

1. Risk of infertility based on an expected treatment plan;
2. Options for fertility preservation (or referral to an appropriate specialist);
3. Documentation of any conversation.

Data also show that patients appreciate having written materials provided to them, as these initial conversations can be overwhelming and patients may not retain all of the information [56,73].

Surgeons may feel that they are underprepared or uneducated regarding infertility discussions, but providing even basic information to patients, making them aware of the risk of infertility, and referring to a specialist can be beneficial (Table 2). In fact, referral to a reproductive specialist emphasizes to patients that fertility is important [48,51], which can help with the cognitive dissonance patients may be feeling about prioritizing non-treatment aspects of their diagnosis [50]. Some studies suggest automatic referrals, as these take the burden off patients about making the decision to see a specialist [46,74]. Additionally, it is important when having these conversations to acknowledge that the prospect of infertility and needing to undergo fertility preservation can add to the stress of a new cancer diagnosis [43], and that the topic may be embarrassing, a feeling that is particularly prevalent among males [43].

When counseling surgical patients in particular about their fertility risks, it is important to acknowledge the unforeseen challenges that these individuals may face, including post-operative complications such as pelvic sepsis that can lead to additional scarring and adhesion formation. Even patients undergoing colon resection alone have risk of these complications, so it is important to discuss fertility with all patients who come in with colorectal cancer, regardless of the location of the primary tumor. Finally, the expected timeline for treatment as well as its urgency should be relayed to patients, as this may give patients the ability to consider fertility preservation without fear of worsening their oncologic outcomes. This is especially relevant in light of recent rectal cancer treatment approaches, where patients may undergo a “watch and wait” approach.

## 6. Conclusions

Infertility is an often overlooked and underdiscussed issue for patients undergoing colorectal cancer treatment. As the incidence of CRC increases among younger adults, who are often focused on establishing their careers and family, the need for discussions regarding infertility are becoming more important. The negative impacts of gonadotoxic chemotherapy and pelvic radiation are well documented, but the role that surgical intervention for CRC plays in infertility is less clear. That said, it has been shown that pelvic surgery, particularly via an open approach, leads to increased adhesion formation, which is known to be associated with female infertility. Furthermore, nerve damage from pelvic surgery has significant implications for ejaculatory issues in males and sexual dysfunction in both males and females, which ultimately impact pregnancy success. Treatment-related infertility places a significant psychological burden on patients, and research shows that patients desire pre-treatment fertility counseling and would take advantage of opportunities for fertility preservation; unfortunately, these conversations occur far too infrequently. Future studies are needed to explicitly elucidate the impact that surgical resection has on fertility in colorectal cancer patients, but as most patients with non-metastatic disease will see a surgeon early on in their treatment course, surgeons are in a prime position to initiate these conversations and place appropriate referrals. Surgeons are vastly underutilized when it comes to fertility discussions, and efforts should be made to enact protocols that ensure fertility conversations are being had with patients in surgical clinics and that patients are being referred to fertility specialists appropriately.

## Figures and Tables

**Table 1:** Fertility consequences of potential injuries secondary to colorectal surgery [17,28,30,31,33,34,35].

Injury/Risk	Fertility-Related Consequence	Time at Risk	Means of Avoidance
*Male*			
Superior hypogastric plexus injury (sympathetic fibers)	Retrograde/absent ejaculation	High ligation of the inferior mesenteric artery	Ligation of the inferior mesenteric artery should be performed 1–2 cm distal to the origin
Hypogastric nerves (sympathetic fibers)	Retrograde/absent ejaculation	Posterior mobilization of the rectum	Careful dissection close to the fascia propria of the rectum; maintain visualization
Pelvic plexus (mixed sympathetic and parasympathetic fibers	Retrograde/absent ejaculation Erectile dysfunction	Lateral mobilization of the rectum	Avoid finger dissection; avoid clamping the middle rectal pedicle; divide lateral ligaments as close to specimen as possible
Cavernous plexus/nervi erigentes (parasympathetic fibers)	Erectile dysfunction	Anterior mobilization of the rectum	Leave prostatic capsule in place
*Female*			
Direct damage to ovaries, fimbriae, or fallopian tubes	Mechanical ovarian dysfunction	Pelvic dissection	Direct visualization during dissection; avoid blunt dissection
Pelvic adhesions	Fallopian tube occlusion; distorted anatomic relation of ovaries, fimbriae, and fallopian tubes; restriction of pelvic blood supply	Pelvic dissection	Laparoscopic as opposed to open procedure, when feasible

**Table 2:** Patient concerns related to infertility counseling and methods to address them in a non-specialist clinic.

Concern	Potential Solution
Lack of control, lack of foresight regarding future infertility	Providers should initiate discussions regarding fertility risk as opposed to waiting for patients to bring up their concerns
Embarrassment and apprehension related to fertility discussions	Acknowledge the uncomfortable nature of these conversations and normalize discussions about fertility Ask patients who they want present during the conversation
Information and decision overload	Provide written materials Automatic referrals to fertility specialists
